# Phospholipids Differentially Regulate Ca^2+^ Binding to Synaptotagmin-1

**DOI:** 10.1021/acschembio.3c00772

**Published:** 2024-04-03

**Authors:** Sophie A. S. Lawrence, Carla Kirschbaum, Jack L. Bennett, Corinne A. Lutomski, Tarick J. El-Baba, Carol. V. Robinson

**Affiliations:** † Department of Chemistry, University of Oxford, South Parks Road, Oxford OX1 3QZ, U.K.; ‡ The Kavli Institute for Nanoscience Discovery, University of Oxford, South Parks Road, Oxford OX1 3QU, U.K.

## Abstract

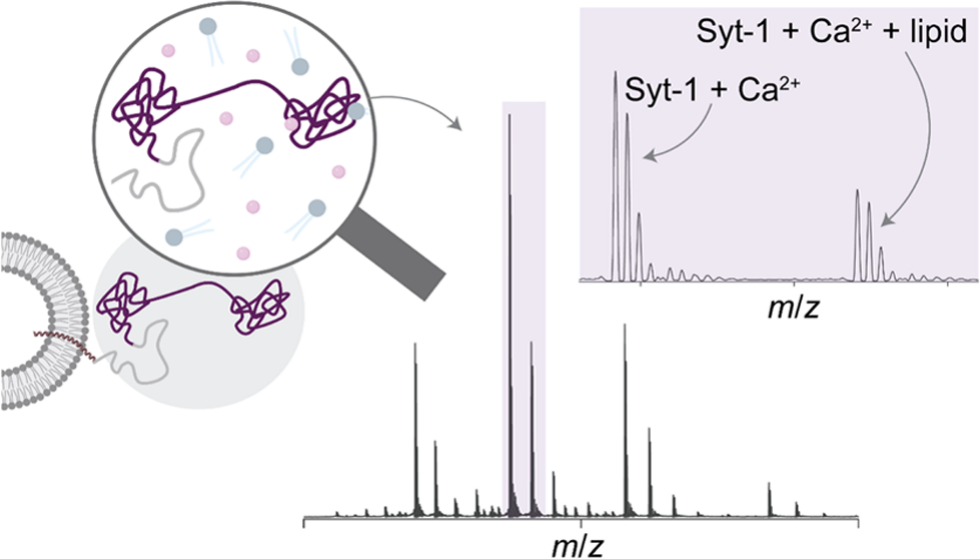

Synaptotagmin-1 (Syt-1) is a calcium sensing protein that is resident in synaptic vesicles. It is well established that Syt-1 is essential for fast and synchronous neurotransmitter release. However, the role of Ca^2+^ and phospholipid binding in the function of Syt-1, and ultimately in neurotransmitter release, is unclear. Here, we investigate the binding of Ca^2+^ to Syt-1, first in the absence of lipids, using native mass spectrometry to evaluate individual binding affinities. Syt-1 binds to one Ca^2+^ with a *K*_D_ ∼ 45 μM. Each subsequent binding affinity (*n* ≥ 2) is successively unfavorable. Given that Syt-1 has been reported to bind anionic phospholipids to modulate the Ca^2+^ binding affinity, we explored the extent that Ca^2+^ binding was mediated by selected anionic phospholipid binding. We found that phosphatidylinositol 4,5-bisphosphate (PI(4,5)P_2_) and dioleoylphosphatidylserine (DOPS) positively modulated Ca^2+^ binding. However, the extent of Syt-1 binding to phosphatidylinositol 3,5-bisphosphate (PI(3,5)P_2_) was reduced with increasing [Ca^2+^]. Overall, we find that specific lipids differentially modulate Ca^2+^ binding. Given that these lipids are enriched in different subcellular compartments and therefore may interact with Syt-1 at different stages of the synaptic vesicle cycle, we propose a regulatory mechanism involving Syt-1, Ca^2+^, and anionic phospholipids that may also control some aspects of vesicular exocytosis.

## Introduction

**Figure 1:**
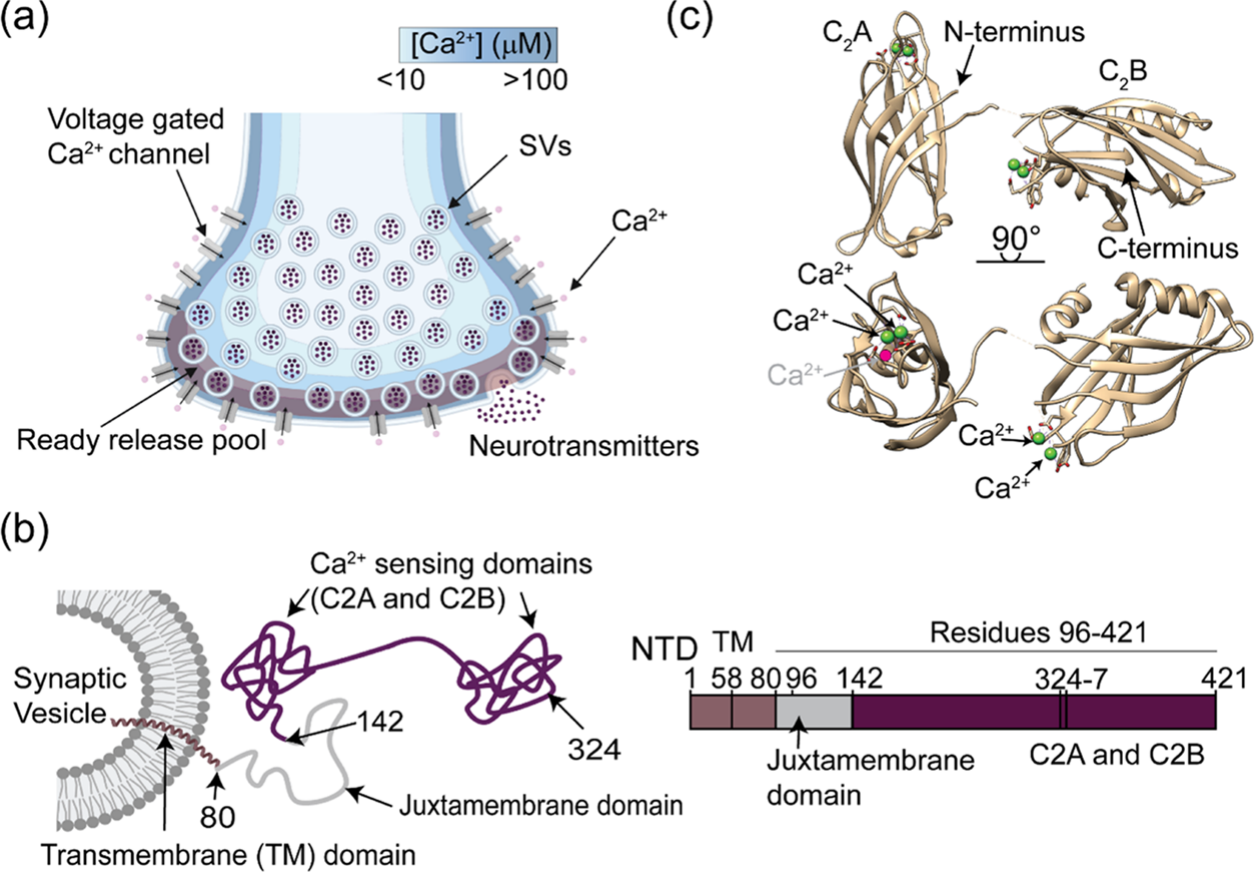
Syt-1 localization and structure. (a) Cartoon depiction of [Ca^2+^] gradient experienced within a presynapse immediately following Ca^2+^ influx. In this simplified model, the concentration gradient of Ca^2+^ decreases from ∼100 μM near the plasma membrane,^4^ to much lower concentrations within the center of the neuron. Syt-1 locations depend on the positions of SVs relative to this gradient. Values adapted from ref^4^. (b) Model of Syt-1 on an SV and overall domain architecture. The construct used in this study consists of residues 96–421, encompassing both Ca^2+^ sensing domains. (c) Structure of Ca^2+^-bound Syt-1 residues 141–421 (PDB 5CCH).^12^ Green spheres depict Ca^2+^; the fifth Ca^2+^, not observed in the structure, is depicted as a red sphere labeled in gray.

Neurons communicate using neurotransmitters stored in synaptic vesicles (SVs) that are released into synapses upon excitation. Arrival of an action potential at a presynaptic dendrite triggers the opening of Ca^2+^ channels which line the plasma membrane.^1^ Upon the opening of these channels, the increased Ca^2+^ diffuses across a dendrite, where it is sensed by SV-bound proteins called Synaptotagmins (Syts),^1−6^ a family of calcium-sensor proteins (Figure 1).^2,7^ There are 17 reported isoforms of Syts in both mice and humans,^8^ with approximately 15 copies of the predominant isoform Synaptotagmin-1 (Syt-1) found on each SV.^9^ Along with their key roles in neurotransmitter exocytosis, Syts are connected to learning and plasticity, and have implications in neurodevelopmental and psychiatric conditions.^10,11^

Syt-1 consists of an N-terminal vesicular region (residues 1–57), a transmembrane domain (residues 58–80), a variable (juxtamembrane) linker resting near the SV membrane (residues 81–142), and two Ca^2+^ sensing domains (C2A and C2B) (residues 143–421) (Figure 1b,c).^13^ The overall structure of C2A and C2B consists of multistranded β-sandwiches. C2A contains two α helices on the periphery, while C2B has four consecutive lysine residues (K324–K327) referred to as a “polylysine patch”, important for binding lipids clustered on the plasma or SV membrane in the absence of Ca^2+^.^5,14,15^ In the process of SV exocytosis Ca^2+^ binding serves different roles,^16−18^ including deforming the plasma membrane by Ca^2+^-dependent penetration by both C2 domains, promotion of vesicle docking and priming,^19,20^ and retrieval of SVs via endocytosis for subsequent neurotransmitter release. Acidic residues at the tips of the β sheets coordinate at least five Ca^2+^ ions in well-characterized binding cavities.^19^ There are three well-characterized binding sites in C2A and two in C2B.^14^ The role of binding of Ca^2+^ to Syt-1 in rapid neurotransmitter release is well established. However, it is challenging to study each individual Ca^2+^ binding event to Syt-1. This is important to understand as fusion of SVs with the plasma membrane is orchestrated by a series of protein–protein interactions, which are thought to be triggered by Ca^2+^-dependent interactions between Syts, other proteins, and phospholipids.^21−23^ Relative to neuronal plasma membranes,^24^ SVs are enriched in phosphatidylserine (PS), a class of anionic phospholipids that comprises ca. 6–10% of the SV lipidome.^25^ In the absence of anionic phospholipid–Syt interactions, the affinity for Ca^2+^ has been shown previously to be relatively low (*K*_D_ ∼ 45 μM).^4^ However, interactions with PS, and other lipids in SVs or plasma membranes are thought to play a role in tuning Syt–Ca^2+^ interactions.^21−24^ Nevertheless, to date, dissecting the multifactorial Ca^2+^- and lipid-binding events has not been possible, ultimately leaving unanswered questions about the role of these interactions in the SV cycle.

Native mass spectrometry (MS) is a technique that has been used extensively to probe solution equilibria.^26,27^ When performed under nondenaturing, buffering conditions, native MS maintains protein tertiary and quaternary structures during the transition into the gas phase. It is well established that protein ions resemble those found in solution,^28^ and native mass spectra provide readouts about the relative amounts of protein–protein and protein–ligand interactions.^29−32^ Cryo-electron microscopy reconstructions of proteins gently landed onto grids *in vacuo* have recently demonstrated that the *in vacuo* structures are nearly identical to those found in solution.^33,34^ Recent work using native MS has provided an understanding of the multitude of assemblies formed during SNARE complex formation^35,36^ and evaluated the extent that divalent metal ions impact coupling to a G-protein coupled receptor.^37^

Here, we use native mass spectrometry to quantitatively evaluate the individual binding strengths of individual Ca^2+^ ions to Syt-1 in the absence of lipids in solution. We utilized a construct consisting of residues 96–421 (Figure 1b), which contains the extracellular, soluble domains of Syt-1. Residues 96–141 include a portion of the juxtamembrane domain, and 142–421 make up both Ca^2+^ sensing domains (C2A and C2B). In line with previous studies,^4^ we find that in the absence of lipids, and under Ca^2+^ concentrations typically experienced by Syt-1 in a neuron, the first binding event is most favorable (*K*_D_ ∼ 45 μM).^38^ Each successive binding event between Ca^2+^ and Syt-1 becomes increasingly less favorable, *K*_D_ > 45 μM. As demonstrated previously, we find that Ca^2+^ binding is enhanced by PS and phosphatidylinositol 4,5-bisphosphate (PI(4,5)P_2_).^21−23^ Interestingly, we find that when bound to other anionic phospholipids, binding between Syt-1 and Ca^2+^ is distinct from that observed with PS and PI(4,5)P_2_, indicating that selected lipids tune Ca^2+^ binding propensities. In the context of the SV cycle, our findings suggest that Ca^2+^ and lipid binding act in synergy to control aspects of neurotransmitter release.

## Results

### Resolving Ca^2+^ Binding to Syt-1

**Figure 2:**
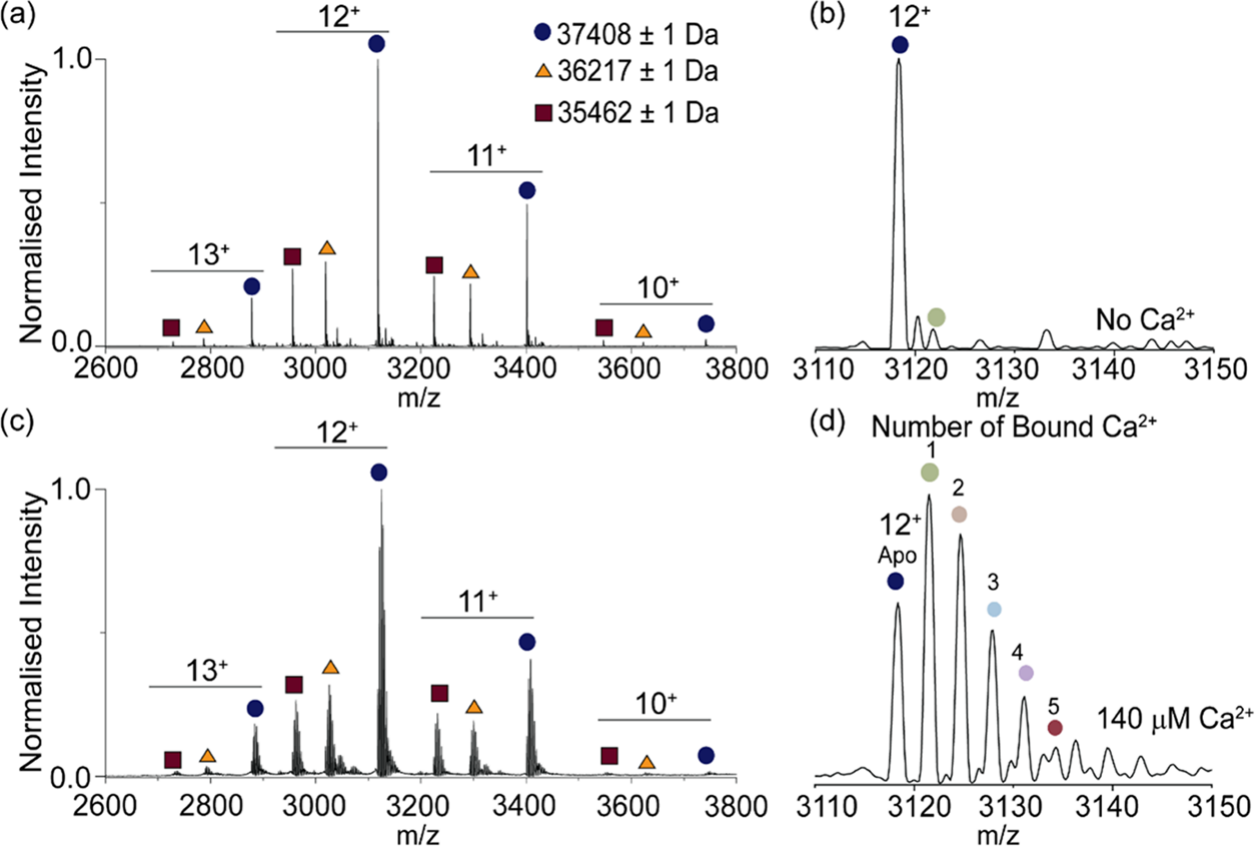
Native mass spectrometry analysis of Syt-1. (a) Native mass spectrum of Syt-1. Measured masses are shown. (b) Detailed view of the 12^+^ charge state to demonstrate that a single binding event is present without exogenous Ca^2+^ addition (dark red circle). (c) Native mass spectrum collected following incubation of Syt-1 (12 μM) with 140 μM Ca(OAc)_2_. (d) Expansion of the 12^+^ charge state demonstrates Ca^2+^ binding event from zero (apo) to five.

To study Ca^2+^ binding to Syt-1, we first generated a native mass spectrum of Syt-1 96–421 (referred to as Syt-1 throughout for simplicity) in 500 mM NH_4_OAc and observed three charge state distributions (Figure 2a); the major distribution corresponds to a protein with molecular mass of 37,408 ± 1 Da. The two minor charge state distributions correspond to proteins with molecular masses of 36,217 ± 1 and 35,462 ± 1 Da (Figure 2a), which are in agreement with N-terminal truncations at residues 104 and 111, respectively (expected masses 36,219 and 35,463 Da) (listed in Table S1). The major distribution was assigned to monomeric Syt-1 (expected mass of 37,410 Da).

We next probed the extent of Ca^2+^ binding by resolving the individual bound states with native MS. Without Ca^2+^ addition, Ca^2+^-bound peaks were observed in each charge state (Figure 2b), indicating that interactions between Syt-1 and endogenous Ca^2+^ survive the purification process. After the incubation of Syt-1 with saturating concentrations of Ca^2+^, we recorded native mass spectra and observed extensive adduction to all charge states of the protein (Figure 2c). It has previously been proposed that Syt-1 can form oligomers on SVs in a Ca^2+^-dependent manner.^26^ So, we explored the possibility of calcium-dependent formation of higher-order structures. We did not observe Ca^2+^-dependent changes in the oligomeric state of Syt-1 (Figure S1), indicating that additional lipids or cofactors might be needed to form these higher-order structures, consistent with previous findings.^22^ Inspection of the charge state distributions for Ca^2+^-treated Syt-1 showed that the adduct peaks are separated by 38 ± 1 Da (Figure 2d), indicating that each Ca^2+^ has replaced two protons on the protein and that we can resolve all five Ca^2+^ binding events.

### *K*_D_ Measurement of Each Ca^2+^ Binding Event

**Figure 3:**
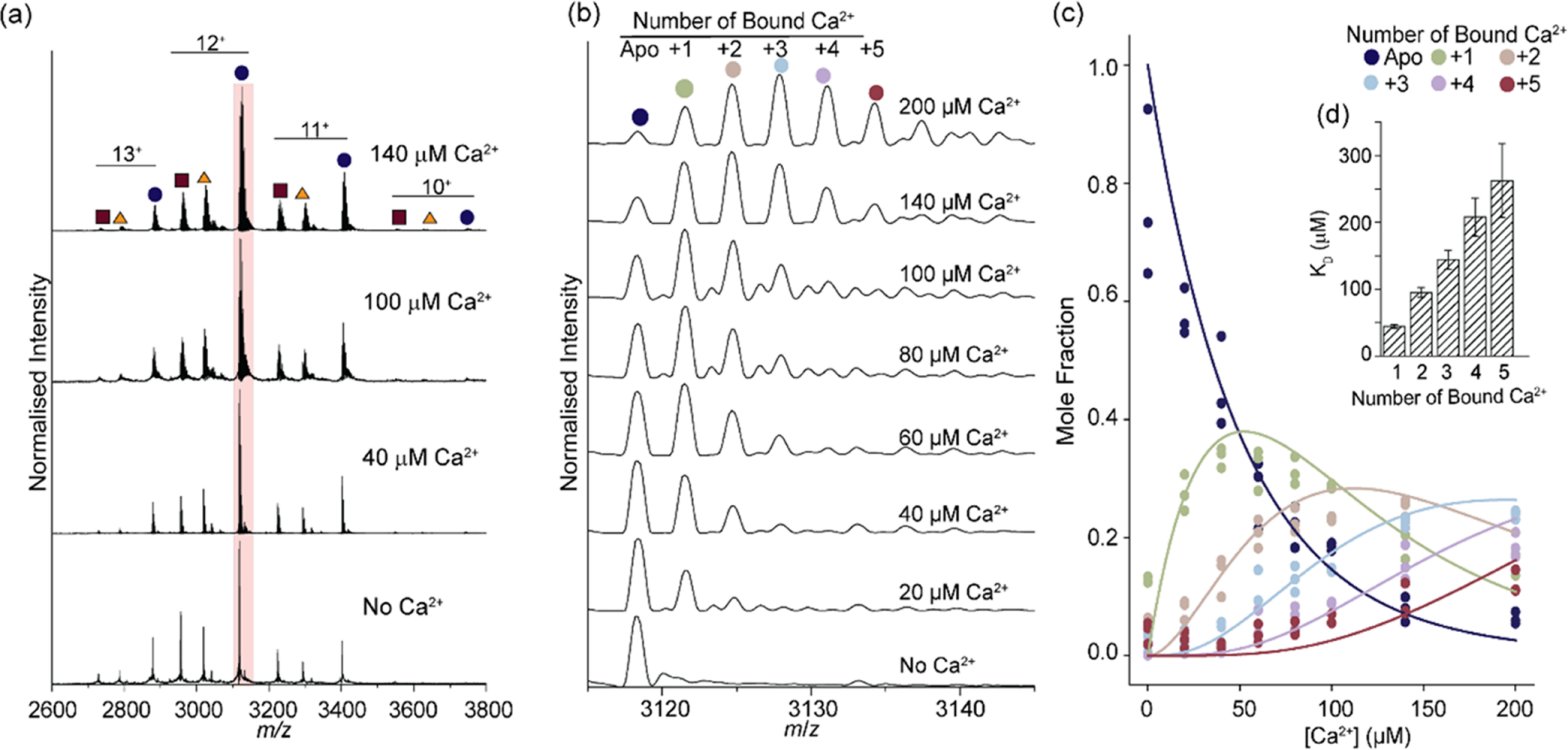
Titration of Ca^2+^ to Syt-1. (a) Stacked native mass spectra at increasing [Ca^2+^] concentrations following EDTA treatment of Syt-1 (12 μM) to remove endogenous binding. The 12^+^ charge state is shaded (pink). (b) Representative native mass spectra of the 12^+^ charge state with increasing [Ca^2+^]. (c) Plot of mole fraction for each Ca^2+^-bound state as a function of total [Ca^2+^]. Solid lines show the fit to an equilibrium binding model to determine the relative binding affinities. Individual measurements from *n* = 3 independent replicates are shown (colored circles). (d) Bar chart to show magnitude of *K*_D_ values for individual Ca^2+^ binding events. *K*_D_ values are reported as mean ± standard deviations, which were derived from an estimated covariance matrix.

Having established that under saturating Ca^2+^ concentrations, individual binding events can be resolved, we next sought to determine binding affinities for each Ca^2+^-bound state. We recorded a native mass spectrum following EDTA treatment to confirm that endogenous Ca^2+^ was depleted (Figure 3a, bottom). Inspection of the 12^+^ charge state of Syt-1 (residues 96–421) showed that predominantly the apo form could be detected, confirming Ca^2+^ depletion (Figure 3b, bottom; Figure S2). We then incubated Syt-1 with increasing [Ca^2+^] and recorded native mass spectra (Figure 3a,b, bottom to top). At low [Ca^2+^] (<40 μM), the most abundant peak is assigned to apo Syt-1. Two additional peaks, each spaced by 38 ± 1 Da, are also present and correspond to one and two bound Ca^2+^ ions, respectively. At a [Ca^2+^] of 40 μM, two binding events were observed; a third feature became clear at a [Ca^2+^] of 60 μM. Interestingly, only at a [Ca^2+^] of 140 μM did we observe evidence for peaks corresponding to four and five bound Ca^2+^ ions. At 200 μM (a 40-fold molar excess), we observed a near-complete loss of the peak corresponding to the apo protein. Together our results show that successive increases in [Ca^2+^] lead to an increase in the extent of Ca^2+^ binding.

To evaluate quantitatively the extent of Ca^2+^ binding across each bound state in Syt-1 (residues 96–421), we plotted the mole fraction of each state (PL*_n_*) as a function of the total [Ca^2+^] (L) (Figure 3c). With an increase in [Ca^2+^], the fraction of unbound Syt-1 decreases, and the fraction of Syt-1 bound to Ca^2+^ increases. As the molar quantity of the first binding event was depleted, there was a subsequent increase in the mole fraction of bound Ca^2+^ adducts (*n* > 1). While higher [Ca^2+^] than those investigated may lead to complete saturation such that the PL_5_ state would dominate, such high [Ca^2+^] are difficult to measure with native MS. Upon incubation of protein solutions with high concentrations of salt (>200 μM) signal suppression, due to undesired peak broadening, is typically observed in native MS measurements.^39^ Moreover [Ca^2+^] > 200 μM are beyond typical physiological limits observed in the presynaptic cell.^40,41^ It is evident however from the plot that even at [Ca^2+^] > 200 μM, not all Syt-1 Ca^2+^ binding sites are saturated (PL_5_); instead, a distribution of bound states is observed.

As the five Ca^2+^ binding events were resolved across the titration, we used an equilibrium binding model to quantify the dissociation constants for sequential binding events.^42−45^ In brief, for multiple binding events, the equilibrium of binding between Syt-1 (P) and Ca^2+^ (L) can be described by a series of equilibrium expressions where *n* is the number of bound Ca^2+^ (and the number of equations required to describe the individual equilibrium constants). In simplified terms
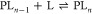
1where *n* = 1, 2,..., 5 in our studies. These equilibrium expressions are readily described in terms of the susceptibility for each PL*_n_* to dissociate, K_D,*n*_
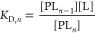
2We determined the individual *K*_D,*n*_ values by globally fitting the model to the experimental data (Figure 3c). After solving these expressions simultaneously, individual *K*_D_ values were obtained for each Ca^2+^ binding event to Syt-1 (*K*_D,1_: 44.1 ± 2.8 μM, *K*_D,2_: 95.3 ± 7.1 μM, *K*_D,3_: 144.2 ± 14.1 μM, *K*_D,4_: 208.0 ± 28.3 μM, *K*_D,5_: 262.4 ± 55.2 μM).

The importance of the *K*_D_’s becomes apparent when comparing the successive values (Figure 3d). With each binding event, the *K*_D_ increases indicating that subsequent Ca^2+^ binding events become successively less favorable. To gain insight into the possible reasons for the sequential reduced Ca^2+^ binding affinity (*viz*., increases in *K*_D_), we inspected the X-ray structures of apo and Ca^2+^-bound Syt-1.^4,5^ The Ca^2+^-free state adopts a conformation in which the two domains interact. In such an arrangement, the empty Ca^2+^ coordination sites in the C2A loops would be filled through hydrogen bonding interactions from side chains in C2B.^46^ It has been hypothesized that the first Ca^2+^ binding event releases these residues.^4,46^ Consistent with our *K*_D_ values, this would unlock the remaining Ca^2+^ binding sites in C2A and C2B such that they sequester Ca^2+^ with roughly the same (or lower) binding affinity. It is therefore reasonable to conclude that at least one binding site will be occupied under physiological Ca^2+^ concentrations following Ca^2+^ influx (concentrations determined as 10–100 μM^40−50^). Moreover, the cytosolic [Ca^2+^] is not expected to rise beyond the highest *K*_D_ value we measured (>200 μM). Full saturation of all Ca^2+^ binding sites *in vivo* is therefore unlikely.

### Lipid Binding Impacts Syt-1/Ca^2+^ Interactions

Since interactions between Syt-1 and anionic phospholipids have been reported to enhance the binding affinity to Ca^2+^,^46^ we compared the impact of different phospholipids on Ca^2+^ binding *K*_D_ values. We opted to investigate phospholipids that have well-established subcellular enrichments: PS an anionic lipid enriched in SV membranes; PI(4,5)P_2_, found exclusively in the plasma membrane; the structural analogues of PI(4,5)P_2_: phosphatidylinositol 3,4-bisphosphate (PI(3,4)P_2_), and phosphatidylinositol 3,5-bisphosphate (PI(3,5)P_2_); and phosphatidylcholine, a positively charged lipid which is the major component of biological membranes. The primary site of PI(3,5)P_2_ synthesis is localized to endosomal and lysosomal membranes,^49^ and PI(3,4)P_2_ is a minor component of the plasma membrane.^50^ As PS and PI(4,5)P_2_ have been reported to directly interact with Syt-1 and positively modulate Ca^2+^ binding,^51^ our study was designed to establish the extent that these lipids fine-tune individual Ca^2+^ binding.

**Figure 4:**
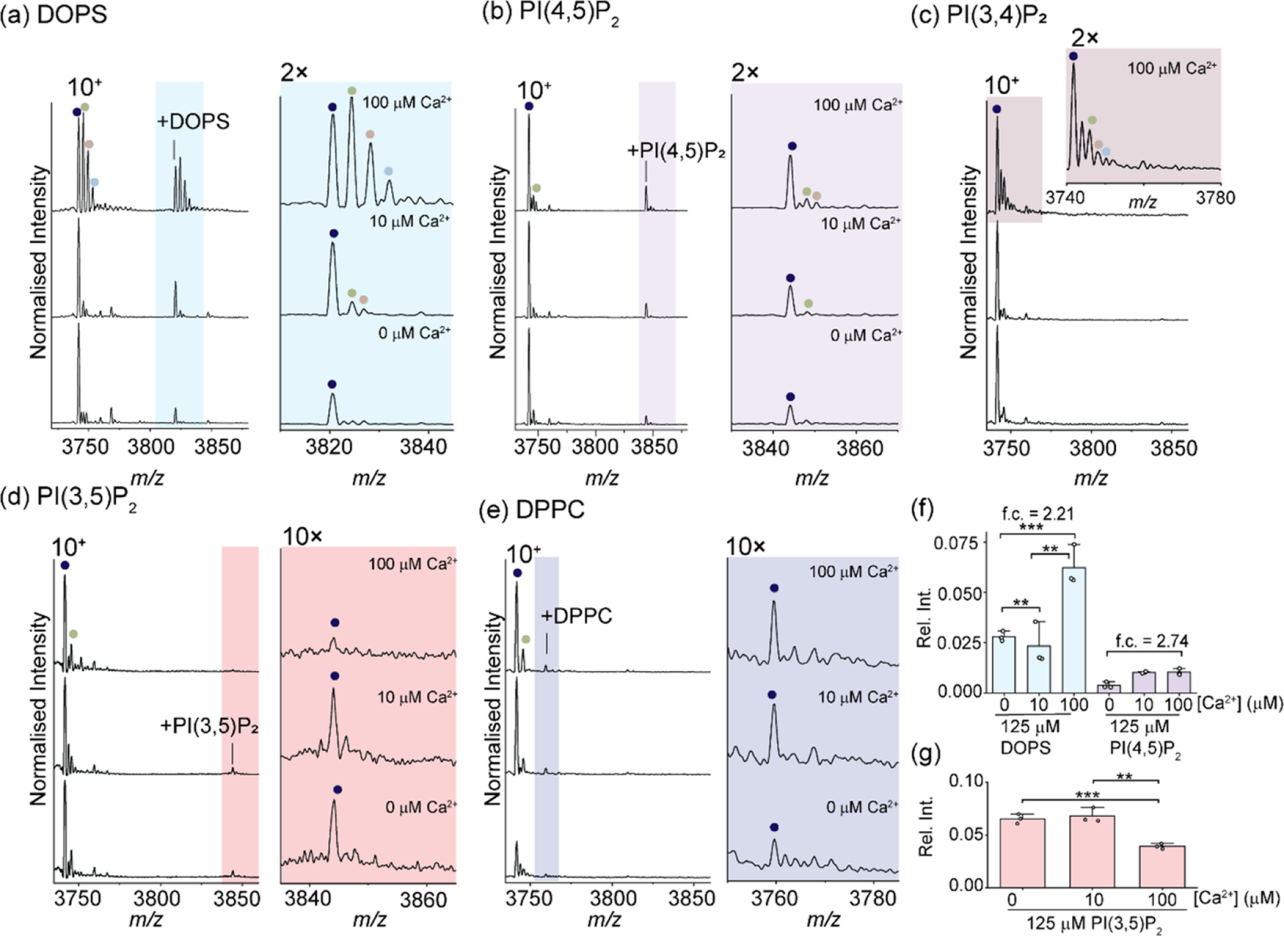
Role of lipids on Ca^2+^ binding to Syt-1. (a) (Left) Representative native mass spectra of Syt-1 (12 μM) in the presence of 125 μM DOPS. (Right) magnified view of the Ca^2+^ and DOPS binding distribution. (b) (Left) Representative native mass spectra of Syt-1 (12 μM) in the presence of 125 μM PI(4,5)P_2_. (Right) Magnified view of the Ca^2+^ and PI(4,5)P_2_ binding distribution. (c) Representative native mass spectra of Syt-1 (12 μM) in the presence of 125 μM PI(3,4)P_2_. (Inset) magnified view to show the Ca^2+^ binding distribution in the presence of 100 μM Ca^2+^. (d) (Left) Representative native mass spectra of Syt-1 (12 μM) in the presence of 125 μM PI(3,5)P_2_. (Right) Magnified view to show the absence of Ca^2+^ and PI(3,5)P_2_ binding. (e) (Left) representative native mass spectra of Syt-1 (12 μM) in the presence of 125 μM DPPC. (Right) Magnified view to show the absence of Ca^2+^ and DPPC binding. (f) Bar plot of the relative intensities of the Syt-1-lipid-Ca^2+^ bound state in the presence of select lipids at different [Ca^2+^]. f.c. = fold change. (g) Bar plot of the relative intensity of Syt-1-PI(3,5)P_2_ at different [Ca^2+^]. Bars represent the average from *n* = 3 independent replicates, and error bars represent the standard deviation. *P ≤ 0.05, **P ≤ 0.01, ***P ≤ 0.001. Deconvoluted mass spectra are shown in Figure S3.

We first recorded native mass spectra of Syt-1 after incubation with dioleylphosphatidylserine (DOPS), a representative lipid from the PS lipid class. We also varied the [Ca^2+^] so that we could explore synergistic binding between the lipid and each Ca^2+^ binding event (Figures 4a and S3a). We note that in the presence of C8E4, a detergent needed for lipid binding experiments,^44^ a shift in the predominant charge state from 12^+^ to 10^+^ was observed. Without Ca^2+^ addition, but at a 10-fold molar excess of DOPS, we observed additional peaks, adjacent to the main charge state distribution, assigned to Syt-1 bound to DOPS (mass addition of 789 Da). After incubating this preparation with 10 or 100 μM Ca^2+^, we observed clear evidence for additional peaks that are characteristic of Ca^2+^ binding to both Syt-1 and DOPS-bound Syt-1 (Figure 4a). However, no changes in the oligomeric state of Syt-1 were observed (Figure S4), likely because higher-order structures reported previously require additional cofactors or conditions to form.^26^ As [Ca^2+^] was increased, more Ca^2+^ adduct peaks were observed (Figure 4a,right). To ensure that the population of all lipid- and Ca^2+^-bound states shifted systematically with varying DOPS concentration, we also varied the amount of DOPS, for each of the three [Ca^2+^] used in the lipid binding experiments (Figure S5a). Indeed, at all [Ca^2+^] with 25 μM DOPS, evidence remained for peaks corresponding to Syt-1 bound to both Ca^2+^ and DOPS.

We next recorded native mass spectra following the incubation of Syt-1-Ca^2+^ mixtures with PI(4,5)P_2_. In the absence of Ca^2+^, at a 10-fold molar excess of PI(4,5)P_2_, an adduct peak corresponding to the lipid was visible in the spectrum (adduct mass of 1023 Da) (Figure 4b, bottom). Upon addition of Ca^2+^ to this complex, an array of peaks corresponding to the Ca^2+^-bound states were observed (Figure 4b, middle, top). Interestingly, at a 2-fold molar excess of PI(4,5)P_2_ (25 μM), there was no evidence of lipid-bound complexes in the native mass spectra (Figure S5b). However, upon addition of Ca^2+^, peaks corresponding to the binding of both PI(4,5)P_2_ and Ca^2+^ to Syt-1 were evident. Furthermore, with higher [Ca^2+^], the abundance of both PI(4,5)P_2_ and Ca^2+^-bound adducts increased concomitantly (Figure 4b, right), indicating synergistic binding.

To identify whether the synergistic binding with PI(4,5)P_2_ was unique relative to other isomers of phosphatidylinositol bisphosphate, we recorded native mass spectra after incubating Syt-1 with a 10-fold molar excess of PI(3,4)P_2_ or PI(3,5)P_2_ (Figure 4c,d), and at a 2-fold molar excess of PI(3,4)P_2_ or PI(3,4)P_2_ (25 μM) (Figure S5c,d). Peaks corresponding to PI(3,4)P_2_ binding were not observed under any of the conditions tested. In contrast, we identified peaks corresponding in mass to binding of PI(3,5)P_2_ to Syt-1 (1026 Da mass adduction) (Figure 4d). However, no Ca^2+^ bound peaks were observed upon titration of Ca^2+^ to this lipid-bound complex; moreover, the PI(3,5)P_2_–Syt-1 adduct peaks were depleted with the addition of Ca^2+^ (Figure 4d, right). This study demonstrates that the interaction between Syt-1 and phosphatidylinositol bisphosphates is finely tuned and specific for the PI(4,5)P_2_ isomer which is uniquely capable of enhancing Ca^2+^ binding.

We further investigated whether ionic phospholipids are key for tuning Ca^2+^ binding by screening the propensity of Syt-1 to interact with dipalmitoylphosphatidylcholine (DPPC), a representative cationic phospholipid and a major component of biological membranes. Surprisingly peaks consistent with binding DPPC to Syt-1 were observed (*viz.*, adduct peaks at +680 Da) (Figures 4e and S5e). However, upon Ca^2+^ addition, no additional peaks corresponding to the Ca^2+^-lipid-bound states were detected (Figure 4e, right) confirming the absence of a synergistic effect of DPPC and Ca^2+^ binding.

To quantitatively evaluate any synergy between lipid and Ca^2+^ binding, we determined the relative amounts of each Ca^2+^ bound state in the presence of the added lipids (Figure 4f). Both the relative fraction of Ca^2+^ binding to the DOPS-bound state and the PI(4,5)P_2_ bound state increased by over 2-fold relative to the Ca^2+^-free conditions. Although we did not observe any Ca^2+^ bound to Syt-1-PI(3,5)P_2_ complexes, we observed a statistically significant depletion of this complex with added Ca^2+^ (Figure 4g). Taken together, our quantitative analysis demonstrates that lipids modulate the Ca^2+^ binding propensities.

If Ca^2+^ binding was tuned by interactions between Syt-1 and any charged membrane lipids, we would anticipate Ca^2+^ binding in the presence of PI(3,4)P_2_, PI(3,5)P_2_, or DPPC. No evidence for binding of these lipids and Ca^2+^ to Syt-1 was detected. These observations allow us to conclude that PI(3,4)P_2_ has no influence on Ca^2+^ binding, as this lipid did not bind in the presence or absence of Ca^2+^. PI(3,5)P_2_ and DPPC on the other hand are unlikely to enhance Ca^2+^ binding since increasing [Ca^2+^] leads to their displacement. Considering the possible location of Syt-1: it can be free in the cell cytosol (unbound to lipid); bound to anionic DOPS on the SV membrane; or bound to PI(4,5)P_2_ on the plasma membrane. In this context, the ability of selected lipids, which are differentially enriched in these subcellular compartments, to tune the Ca^2+^ binding propensities suggests that Syt-1 localization is an important factor to consider when interpreting synergistic binding.

## Discussion

We studied Ca^2+^ binding events to Syt-1 by resolving individual Ca^2+^-bound states with native mass spectrometry. The *K*_D_ for the first Ca^2+^ binding event is ∼45 μM, closely similar to values reported previously.^4^ Successive Ca^2+^ binding affinities were found to be less favorable, a hallmark of negative cooperativity. Binding of Syt-1 to DOPS or PI(4,5)P_2_ leads to an enhancement in the binding affinity for Ca^2+^. Interestingly, and in contrast to Ca^2+^ binding in the absence of lipids, these Syt-1–lipid complexes promote the binding of successive Ca^2+^, prompting the proposal that lipids modulate Syt-1-Ca^2+^ interactions.

The enhancement in affinity for Ca^2+^ can be reconciled by considering the structural impacts of lipid binding. Ca^2+^ is known to bind to cup-shaped cavities in the C2 domains of Syt-1, and the coordination sphere of at least one of these Ca^2+^ binding pockets is known to be incomplete.^51^ Polar side chains are unable to interact to fulfill the coordination sphere, materializing in a weak binding affinity to multiple Ca^2+^ ions.^51^ DOPS and PI(4,5)P_2_ readily bind Syt-1 and multiple Ca^2+^ ions, an indication that the headgroups of these anionic phospholipids complete the empty Ca^2+^ coordination spheres.^51^ Conversely, the structural isomers of PI(4,5)P_2_—PI(3,4)P_2_ and PI(3,5)P_2_—were unable to form a stable complex with both Syt-1 and Ca^2+^. PI(3,4)P_2_ did not bind to Syt-1, and while PI(3,5)P_2_–Syt-1 complexes were detected, they readily disassembled with added Ca^2+^. Therefore, these observations suggest that orientation of the phosphate groups on PIP_2_ isomers plays different roles. Phosphorylation of the fifth position of the inositol backbone is important for PIP_2_ binding.^52^ Ca^2+^-induced disassembly of PI(3,5)P_2_–Syt-1 complexes suggests that the PIP_2_ phosphorylation site at position four, but not five, is critical for completing the empty Ca^2+^ coordination sphere. Given that DOPS and PI(4,5)P_2_ enhance the binding affinity for successive Ca^2+^ ions, these phospholipids are likely important for completing the coordination sphere(s).

**Figure 5:**
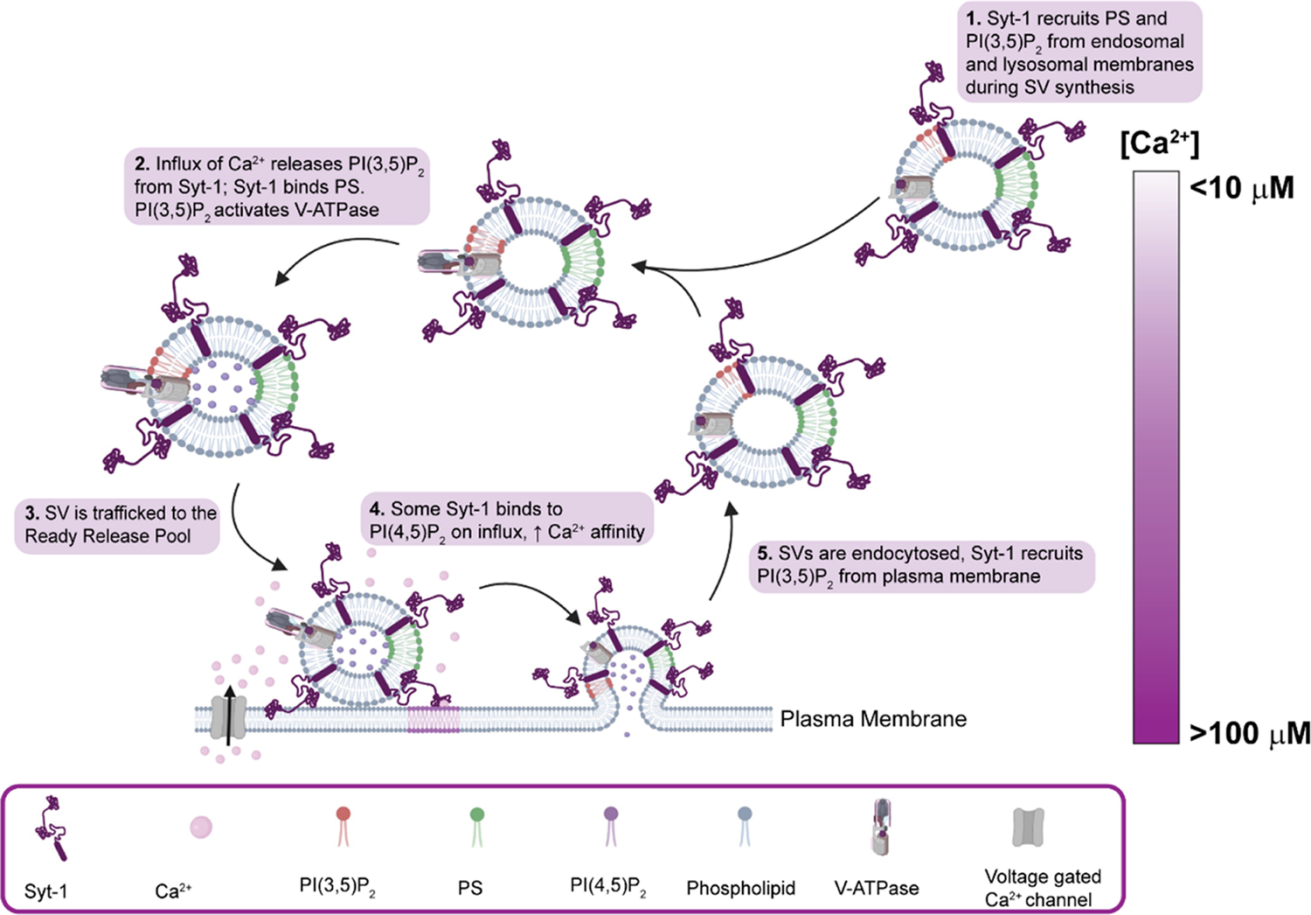
Lipid binding regulates Ca^2+^ binding to Syt-1. Hypothetical model depicting the key roles of PI(3,5)P_2_ and PI(4,5)P_2_ binding to Syt-1. PI(3,5)P_2_ is sequestered from membranes by Syt-1 for the assembly of V-ATPase in the absence of Ca^2+^. Upon influx, PI(3,5)P_2_ is released, and PI(4,5)P_2_ binds to Syt-1 for the release of neurotransmitters.

The binding of PI(3,5)P_2_ and Ca^2+^-dependent disassembly is intriguing in the context of the SV cycle (Figure 5). PIP_2_ isomers overall are low abundance (<0.1%) signaling lipids enriched in the plasma membrane, and have well-established roles in vesicular maintenance.^50,53,54^ The loss of binding between Syt-1 and PI(3,5)P_2_ indicates that it is unlikely to play a central role in fast synchronous neurotransmitter exocytosis. By contrast, this lipid potentially plays a role in SV endocytosis. Studies of the yeast V-type ATPase have found that PI(3,5)P_2_ is required for the assembly of V_1_ and V_0_ to form the V-type ATPase,^55^ a protein complex that acidifies SV thereby priming them for subsequent loading of neurotransmitters by neurotransmitter transporters (Figure 5).^56,57^ It is feasible that Syt-1 binds to and recruits PI(3,5)P_2_ to SVs under low Ca^2+^ conditions. According to our data, Syt-1 releases the captured lipid at high Ca^2+^ concentrations, analogous to an action potential influx. Releasing this lipid would ensure that newly formed SVs, which do not comprise the ready release pool, could promote the assembly of the V-type ATPases.^55^ This permits the acidification of SVs primed for filling with neurotransmitters.

More generally, this application of native mass spectrometry, which links directly the impact of lipid binding on Ca^2+^ binding propensity, reveals an intricate control mechanism that relies in part on subtle differences of isomeric forms of PIP_2_. This native mass spectrometry approach is therefore likely to be broadly applicable when protein–ligand binding is linked to PIP_2_ isomers. Since these isomers are located in membranes that define different subcellular locations,^58^ we envisage further protein ligand studies wherein binding affinities are attenuated by PIP_2_ isomers thereby implying differential regulation within specific membrane compartments.

## Experimental Section

### Reagents

1,2-Dioleoyl-*sn*-glycero-3-phospho-l-serine (sodium salt) (18:1 DOPS), 1,2-dioleoyl-*sn*-glycero-3-phospho-(1′-myo-inositol-4′,5′-bisphosphate) (ammonium salt) (18:1 PI(4,5)P_2_), and 1,2-dioleoyl-*sn*-glycero-3-phosphocholine (18:1 DPPC) were purchased from Avanti Polar Lipids. 1,2-Dioleoyl-*sn*-glycero-3-phospho-(1′-myo-inositol-3′,4′-bisphosphate) (ammonium salt) (18:1 PI(3,4)P_2_) and 1,2-dioleoyl-*sn*-glycero-3-phospho-(1′-myo-inositol-3′,5′-bisphosphate) (ammonium salt) (18:1 PI(3,5)P_2_) were purchased from Merck Millipore. C8E4 was purchased from Anatrace. All other chemicals were purchased from Merck Millipore.

### Protein Expression and Purification

A plasmid encoding rat Syt-1 domains 96–421 was obtained from Addgene (Plasmid 170643, a kind gift from Prof. Ed Chapman’s group). Syt-1 was purified essentially as described.^26^ Briefly, the plasmid was transformed into *Escherichia coli* BL21(DE3) cells and grown overnight on a Luria–Bertani (LB) agar plate supplemented with 100 μg mL^–1^ ampicillin. The following evening, 5–10 colonies were used to inoculate 100 mL of LB broth containing 100 μg mL^–1^ ampicillin and grown overnight (37 °C, 200 rpm). ∼10 mL of the overnight culture was used to inoculate 1 L of LB (100 μg mL^–1^ ampicillin) and grown at 37 °C until an OD_600_ value of 0.6–0.7 was reached. Protein expression was induced with 0.5 mM isopropyl β-d-1-thiogalactopyranoside (IPTG). Cells were harvested (5500*g*, 10 min, 4 °C) after expression overnight at 18 °C. The cells were stored at −80 °C until lysis.

Cells were thawed and resuspended in lysis buffer (20 mM HEPES, 150 mM NaCl) supplemented with EDTA-free protease inhibitor tablets (Roche) before lysis using a microfluidizer. The lysate was clarified by centrifugation (20 min, 20,000*g*, 4 °C) and filtered through a 0.22 μm filter. A gravity column was loaded with 2 mL of GST resin and washed with 25 mL of Milli-Q water followed by 25 mL of lysis buffer. The GST resin was added to the supernatant, and the mixture was left stirring at 4 °C overnight. The following day, the resin was collected using a gravity column and washed with 100 mL of lysis buffer. 250 units of thrombin and 50 mL of cleavage buffer (10 mM KCl, 25 mM HEPES, 5% glycerol) were added to the resin and left to stir overnight at 4 °C. The sample was eluted with 10 mM KCl, 25 mM HEPES, 5% glycerol, and 10 mM reduced glutathione. A 1 mL benzamidine column was used for thrombin removal using the manufacturers’ recommended protocol. Briefly, the column was equilibrated on the AKTA Pure with Milli-Q water and 50 mL of lysis buffer. The sample was loaded, and the flow through was collected in 1 mL fractions. Peak fractions were collected. Syt-1 (∼4.6 mg mL^–1^) was aliquoted and flash frozen in liquid nitrogen and stored at −80 °C.

### Native Mass Spectrometry

Samples were thawed on ice before being exchanged with a buffer in 500 mM ammonium acetate using BioSpin-6 (BioRad) columns. Capillaries were prepared in-house using a P97 Micropipette Puller (Sutter Instrument Corporation) and gold-plated by an Agar Auto Sputter Coater. ∼2.5 μL of protein solutions was loaded into the gold-coated capillaries for nanoelectrospray analysis. MS data was acquired on a Q Exactive mass spectrometer (Thermo Fisher Scientific). The instrument parameters were optimized to maintain native complexes. Collision energy was carefully optimized to permit micelle removal while limiting noncovalent complex dissociation.^59^ Typical instrument parameters were optimized between: in source trapping 10–75 V, typically 50 V; HCD energy 10 V; capillary temperature 150 °C; pressure setting 6; and resolution of the instrument 12,500.

For Ca^2+^ binding experiments, Syt-1 (5.6 μM) and Ca(OAc)_2_ (dissolved in 500 mM ammonium acetate) were combined and allowed to incubate on ice for at least 10 min before being introduced into the mass spectrometer.^45^ For Ca^2+^ depletion by EDTA treatment, Syt-1 (12 μM) and EDTA (20 μM) were incubated on ice for 45 min before excess EDTA was removed using a BipSpin-6 (BioRad) column. For lipid binding experiments, Syt-1 (12 μM) and Ca(OAc)_2_ were combined with lipids in 500 mM ammonium acetate with 2× critical micelle concentration of C8E4 detergent as described previously.^40,60^

### Data Analysis

All MS data was processed using Xcalibur (version 4.3), OriginPro 2023, and Python. Titration data were fit with a sequential binding model with slight changes made to previously described methods.^45^ Notably, to calculate mole fractions as a function of the total Ca^2+^ concentration (rather than the free ligand concentration), equilibrium populations were calculated from kinetic simulations of the system. *K*_D_ values are reported as mean ± s.d., where standard deviations were derived from an estimated covariance matrix and are plotted as error bars in Figure 3d. *P*-values were determined using a two-sampled *t* test in OriginPro 2023. The *K*_D_ values for Ca^2+^ binding were not determined for the truncations.

## Supporting Information Available

Expected and measured molecular weights of Syt-1 and the truncations; native mass spectra at high *m*/*z* depicting the absence of high-molecular-weight Syt-1 oligomers following Ca^2+^ and lipid treatment; deconvoluted mass spectra of lipid- and Ca^2+^-bound Syt-1, and native mass spectra and quantitative analysis of Syt-1-lipid binding experiments at 25 μM lipid (PDF)
