# Supplementary material for: Phospholipids Differentially Regulate Ca2+ Binding to Synaptotagmin-1
Source: ACS Chem Biol. 2024 Apr 3;19(4):953–61. doi: 10.1021/acschembio.3c00772 (PMC11040605; doi:10.1021/acschembio.3c00772)
Supplement: Supplementary file 1 — cb3c00772_si_001.pdf [file cb3c00772_si_001.pdf]

Supporting Information for:

Phospholipids differentially regulate Ca^{2+} binding to Synaptotagmin-1

Sophie A. S. Lawrence,[1,2] Carla Kirschbaum,[1,2] Jack L. Bennett,[1,2] Corinne A. Lutomski,[1,2] Tarick J. El-Baba,[1,2]* and Carol. V. Robinson[1,2]*

1. Kavli Institute for Nanoscience Discovery, University of Oxford, Dorothy Crowfoot Hodgkin Building, University of Oxford, South Parks Road OX1 3QU, U.K.

2. Department of Chemistry, University of Oxford, South Parks Road, OX1 3QU, U.K.

*Correspondence to: tarick.el-baba@chem.ox.ac.uk or carol.robinson@chem.ox.ac.uk

Table S1. Expected and Measured Masses of the three lengths of Syt-1

**Residue Length**	**Expected Mass (Da)**	**Measured Mass (Da)**
96-421	37,410	37,408 ± 1
104-421	36,219	36,217 ± 1
111-421	35,463	35,462 ± 1

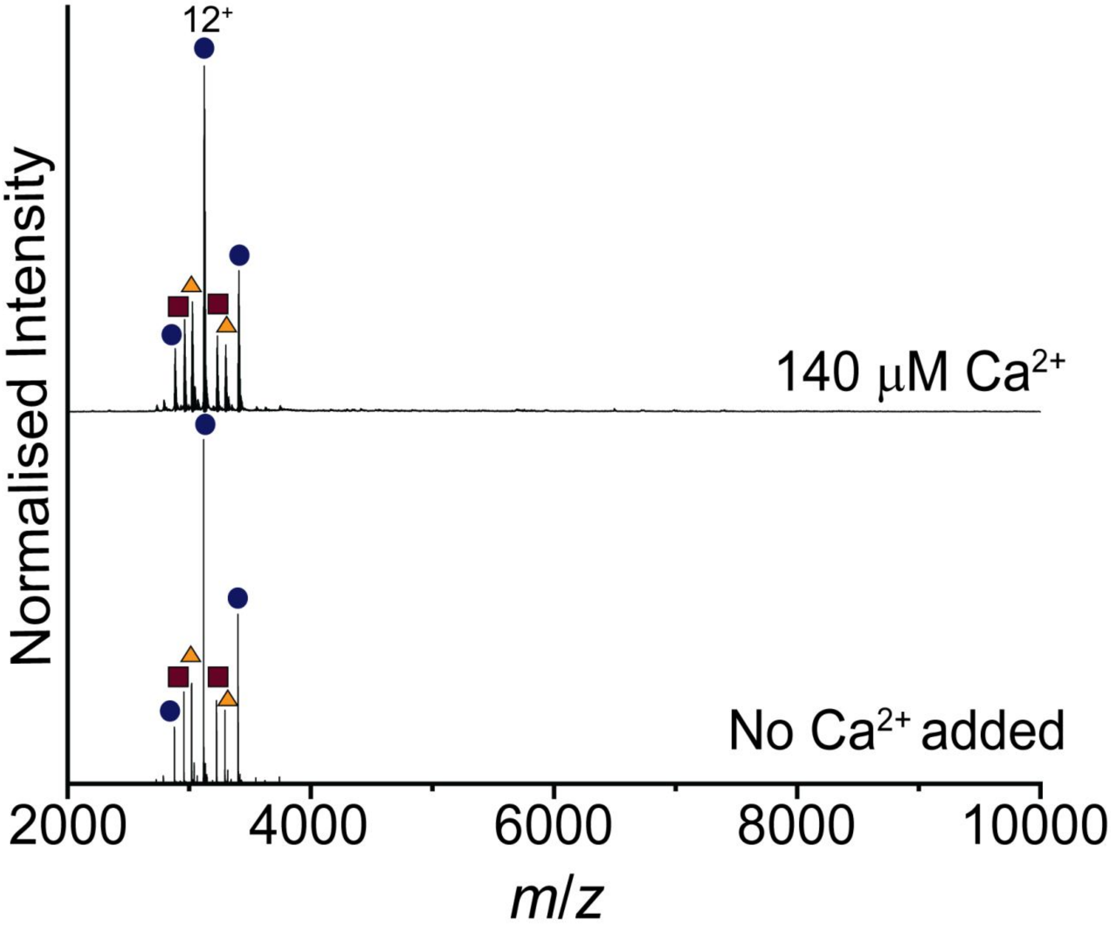

Figure S1. ***Synaptotagmin-1 oligomers are not detected upon binding to*** Ca^{2+}**.** Native mass spectra of Synaptotagmin-1 in the presence of 140 μM $Ca(OAc)_2$ (top) and in the absence of exogenous Ca^{2+} (bottom). No higher *m*/*z* peaks, consistent with oligomer formation, were observed.

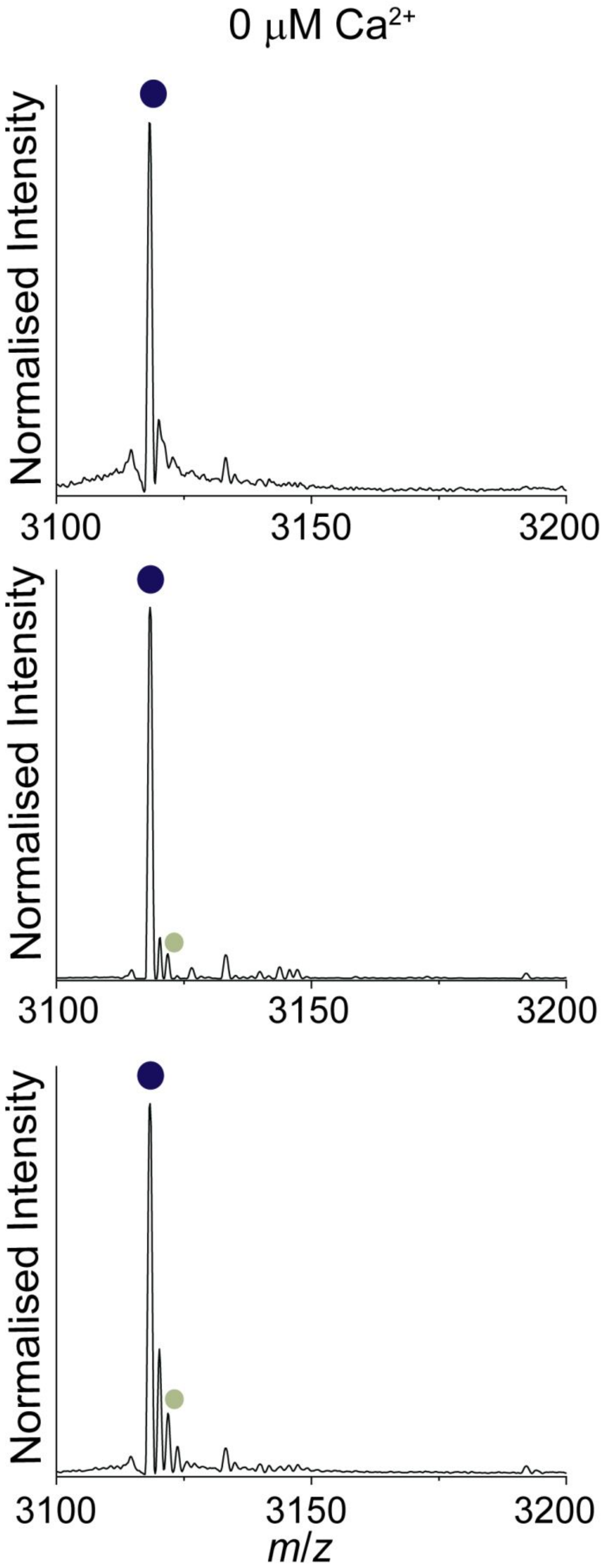

Figure S2. Native mass spectra of Syt-1 upon EDTA treatment. 12^{+} charge state of Syt-1 (12μM) upon EDTA treatment is shown for each independent replicate.

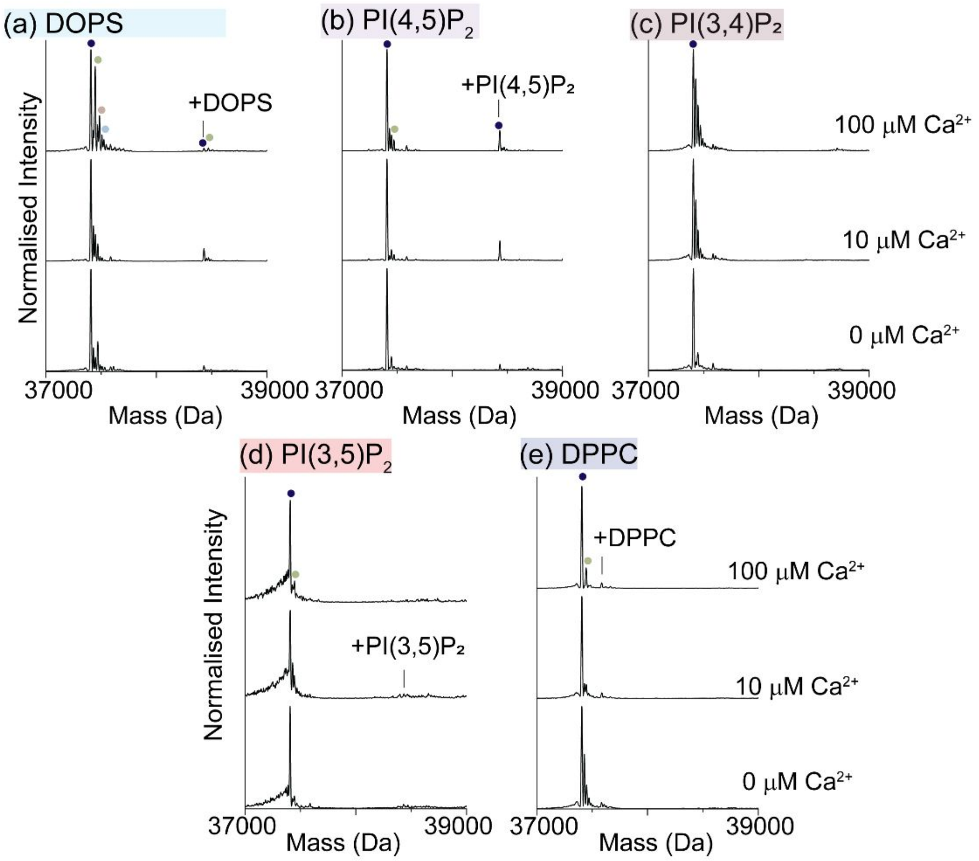

Figure S3. Deconvolved Mass Spectra of Syt-1 in the presence of Lipids. (a) Deconvolved mass spectra of Syt-1 (12 μM) in the presence of 125 μM DOPS. **(b)** Deconvolved mass spectra of Syt-1 (12 μM) in the presence of 125 μM PI(4,5)P_2. **(c)** Deconvolved mass spectra of Syt-1 (12 μM) in the presence of 125 μM PI(3,4)P_2. **(d)** Deconvolved mass spectra of Syt-1 (12 μM) in the presence of 125 μM PI(3,5)P_2. **(e)** Deconvolved mass spectra of Syt-1 (12 μM) in the presence of 125 μM DPPC.

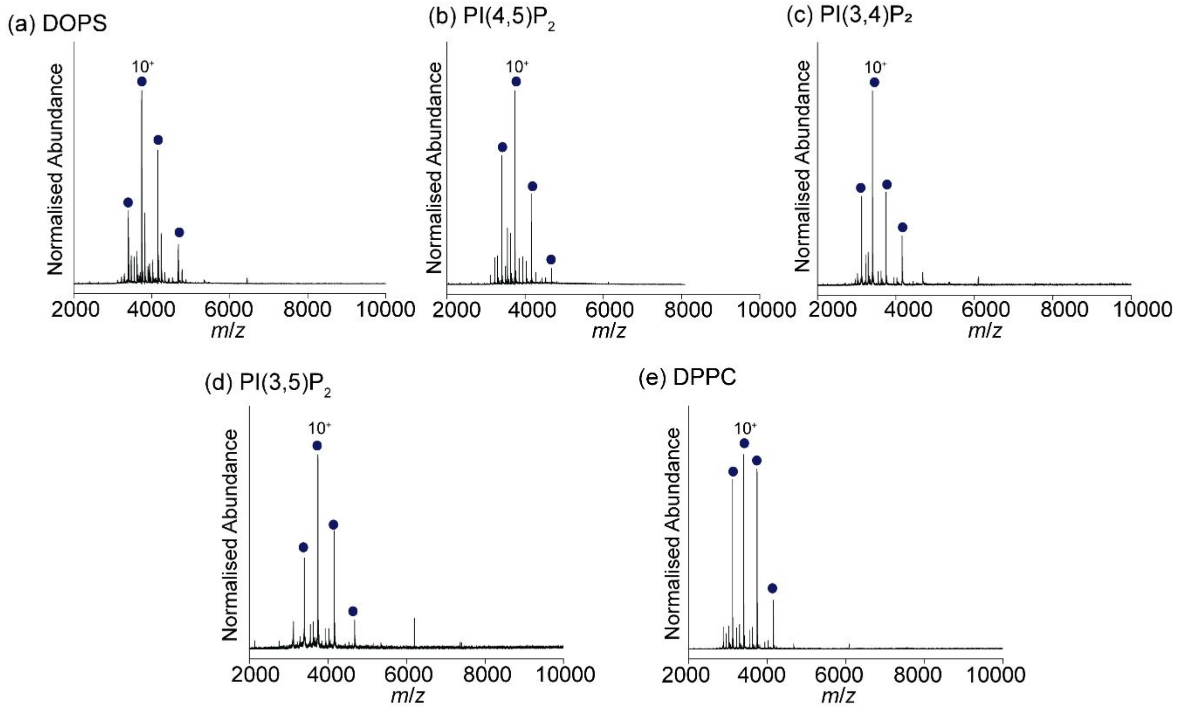

Figure S4. Synaptotagmin-1 oligomers are not observed upon binding to lipids and Ca^{2+}. Native mass spectra of Synaptotagmin-1 in the presence of 100 μM $Ca(OAc)_2$ and in the presence of 125 μM: (a) DOPS, (b) $PI(4,5)P_2$, (c) $PI(3,4)P_2$, (d) $PI(3,5)P_2$, (e) DPPC. No higher *m*/*z* peaks, consistent with oligomer formation, were observed.

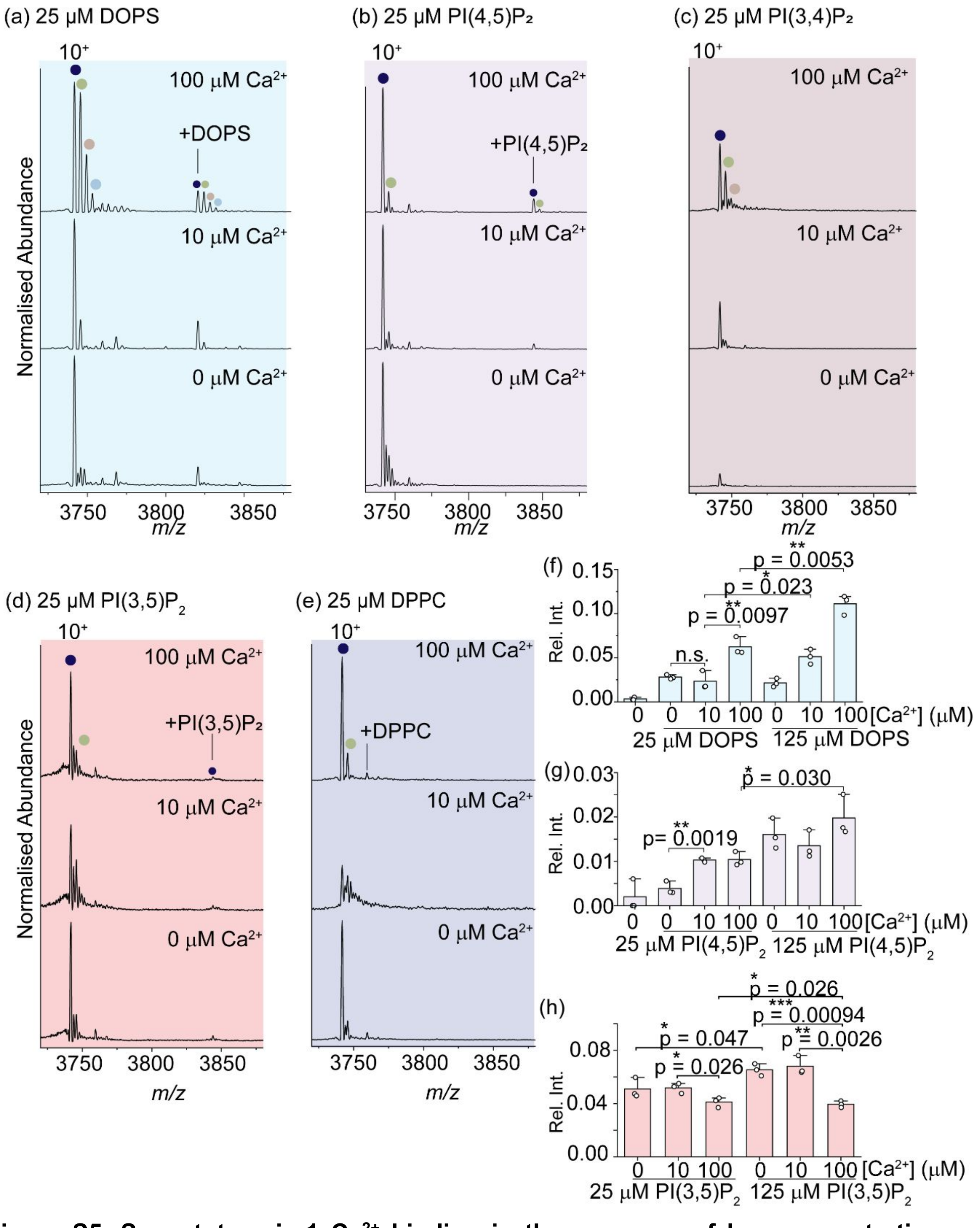

Figure S5. Synaptotagmin-1 Ca^{2+} binding in the presence of low concentrations of lipids. Representative native mass spectra of Syt-1 (12 μM) in the presence of **(a)** 25 μM DOPS **(b)** 25 μM PI(4,5)P_2 **(c)** 25 μM PI(3,4)P_2 **(d)** 25 μM PI(3,5)P_2 **(e)** 25 μM DPPC. Increasing [Ca^{2+}] enhances Ca^{2+} and lipid binding for DOPS and PI(4,5)P_2. No lipid binding seen for PI(3,4)P_2 and increasing [Ca^{2+}] reduces lipid binding for PI(3,5)P_2. **(f)** Bar plot of the relative intensities of the Syt-1-DOPS-Ca^{2+} bound state at different [Ca^{2+}] and [DOPS]. **(g)** Bar plot of the relative intensities of the Syt-1-PI(4,5)P_2-Ca^{2+} bound state at different [Ca^{2+}] and [PI(4,5)P_2]. f.c. = fold change. **(h)** Bar plot of the relative intensity of Syt-1-PI(3,5)P_2 at different [Ca^{2+}] and [PI(3,5)P_2]. Bars represent the average from n=3 independent replicates and error bars represent the standard deviation. * $P \leq 0.05$, ** $P \leq 0.01$, *** $P \leq 0.001$.
